# Synthesis and characterization of gold(I) thiolate derivatives and bimetallic complexes for HIV inhibition

**DOI:** 10.3389/fchem.2024.1424019

**Published:** 2024-07-25

**Authors:** Christian K. Adokoh, Akwasi Boadu, Isaac Asiamah, Clement Agoni

**Affiliations:** ^1^ Department of Forensic Sciences, School of Biological Sciences, College of Agriculture and Natural Sciences, University of Cape Coast, Cape Coast, Ghana; ^2^ Discipline of Pharmaceutical Sciences, School of Health Sciences, University of KwaZulu-Natal, Durban, South Africa; ^3^ Wesbury College of Science, KwaZuluNatal, South Africa; ^4^ Department of Chemistry, School of Physical Sciences, College of Agriculture and Natural Sciences, University of Cape Coast, Cape Coast, Ghana

**Keywords:** anti-retroviral treatments, computer-aided drug design, gold(I) thiolate derivatives, bimetallic complexes, molecular docking, molecular dynamics, human immunodeficiency virus protease inhibitors, darunavir

## Abstract

**Introduction:** The human immunodeficiency virus (HIV) remains a significant global health concern, with a reported high infection rate of 38.4 million cases globally; an estimated 2 million new infections and approximately 700,000 HIV/AIDS-related deaths were reported in 2021. Despite the advent of anti-retroviral therapy (ART), HIV/AIDS persists as a chronic disease. To combat this, several studies focus on developing inhibitors targeting various stages of the HIV infection cycle, including HIV-1 protease. This study aims to synthesize and characterize novel glyco diphenylphosphino metal complexes with potential HIV inhibitory properties.

**Method:** A series of new gold(I) thiolate derivatives and three bimetallic complexes, incorporating amino phosphines and thiocarbohydrate as auxiliary ligands, were synthesized using procedures described by Jiang, et al. (2009) and Coetzee et al. (2007). Structural elucidation and purity assessment of the synthesized compounds (**1**–**11**) were conducted using micro-analysis, NMR, and infrared spectrometry.

**Results and Discussion:** Using molecular modeling techniques, three of the metal complexes were identified as potential HIV protease inhibitors, exhibiting strong binding affinity interactions with binding pocket residues. These inhibitors demonstrated an ability to inhibit the flexibility of the flap regions of the HIV protease, similar to the known HIV protease inhibitor, darunavir. This study sheds light on the promising avenues for the development of novel therapeutic agents against HIV/AIDS.

## Introduction

Communicable diseases represent significant global challenges, impacting human security, exacerbating poverty, and fostering economic instability (Beagleholeet al., 2011). Among these diseases, the human immunodeficiency virus (HIV) is reported to have a high infection rate, with a global prevalence of 38.4 million in 2021, and there were an estimated 2 million new infections and approximately 700 000 HIV/AIDS-related deaths in the same year (UNAIDS and Sheet, 2021). Despite advances in treatment, HIV/AIDS remains a chronic condition, necessitating ongoing efforts to develop novel therapeutic approaches (Levitt et al., 2011).

Currently, the treatment of HIV/AIDS involves combination antiretroviral therapy (cART) (Ghosh, 2023), non-nucleoside reverse transcriptase inhibitors (NNRTIs), the fusion inhibitor enfuvirtide (T-20) (Ghosh, 2023), and gene editing platforms based on the clustered regularly interspaced short palindromic repeat–Cas system (CRISPR-Cas) (Hussein et al., 2023).

HIV protease is among the biomolecules targeted in the design of HIV therapeutics (Arrigoni et al., 2023). It is involved in the cleavage of viral polyproteins, leading to viral protein maturation (Chuntakaruk et al., 2024). Therapeutically blocking HIV protease therefore halts viral maturation, thus presenting HIV protease as a prominent target for HIV drug discovery efforts. Several HIV protease inhibitors have been developed over the years (King et al., 2004; Ghosh et al., 2006; Chuntakaruk et al., 2024). Among these is darunavir, whose binding is characterized by strong interactions between the 3(R),3a(S),6a(R)-bis-tetrahydrofuranyl (bis-THF) moiety of darunavir and the main-chain atoms of aspartates 29 and 30 (King et al., 2004; Ghosh et al., 2006; Chuntakaruk et al., 2024).

Advancements in metal-based antiviral therapeutics, particularly involving gold (Au) and silver (Ag), have shown promise in enhancing safety and minimizing side effects (King et al., 2004; Ghosh et al., 2006). Metal–drug synergism, where drug stability is enhanced by coordination with metal ions, offers advantages such as improved pharmacokinetics and drug metabolism (Pettersen et al., 2004; Trott and Olson, 2010; ChemAxon, 2013; Kožíšek et al., 2014). Gold-based therapeutics, in particular, have demonstrated efficacy in inhibiting viral entry by interacting with Gp120 and CD4 (Hanwell et al., 2012).

Despite uncertainties surrounding the precise mechanisms of action, studies have shown that gold compounds effectively block viral entry and inhibit HIV proteases (Hanwell et al., 2012; Le Grand et al., 2013). Notably, gold(I) phosphine compounds have exhibited inhibitory activity against HIV-1 reverse transcriptase (RT) and protease (PR) without causing toxicity to immune cells (Le Grand et al., 2013). Metal–carbohydrate compounds have also shown potential in reducing toxicity and enhancing biocompatibility, offering promising avenues for therapeutic development (Ramharack et al., 2023). However, concerns regarding side effects, such as skin discoloration upon light exposure, have been reported with gold-based therapeutics (Agoni et al., 2018a).

Although antiretroviral therapy (ART) has extended the lifespan of HIV patients, there remains a need for therapeutics capable of effectively inhibiting HIV-infected cells (Olotu et al., 2019). To address this need, pharmaceutical companies are increasingly leveraging computer-aided techniques to predict drug candidates, reducing research costs and failures attributed to poor pharmacokinetic properties (Agoni et al., 2022). *In silico* molecular docking, molecular simulation, and pharmacokinetic analyses offer valuable insights into ligand–target protein interactions, aiding in the identification of potential therapeutic agents (Salomon-Ferrer et al., 2013; Maier et al., 2015).

In this study, we seek to synthesize and characterize novel glyco diphenylphosphino metal complexes with the potential to combat HIV. Leveraging advanced molecular modeling techniques, we aim to elucidate the mechanisms underlying the anti-HIV activities of these compounds at a molecular level. By unraveling these intricate interactions, we seek to contribute to the development of more effective therapeutic strategies against HIV.

## Methodology

### Materials and method

**FIGURE 1:**
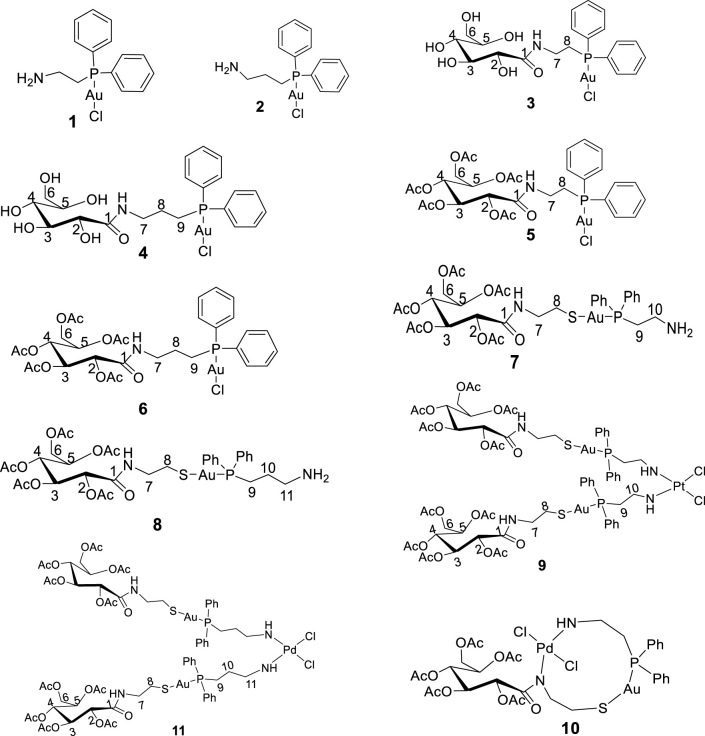
Structure of new compounds (**1–11**) designed and synthesized in this study.

Toluene, dichloromethane, and methanol, used as solvents, were dried using an SPS-1 stand-alone solvent purifier distillation system over standard reagents under N_2 (g)_ prior to use. D-(+)-Gluconic acid δ-lactone, tetrahydrothiophene, 2-(diphenylphosphino)ethanamine, 3-(diphenylphosphino)propanamine, acetic anhydride, hydrogen tetrachloroaurate trihydrate, dimethylaminopyridine (DMAP), and triethylamine were purchased from Sigma-Aldrich and used without further purification. [(tht)AuCl] (tht = tetrahydrothiophene), 2-gluconamido ethane thiol, and acetylated 2-gluconamido ethane thiol were prepared according to previous procedures (Coetzee et al., 2007; Jiang, et al., 2009). The proposed novel compounds synthesized and investigated virtually are given in Figure 1.

### Instrumentation

All NMR spectra of CD_3_OD and CDCl_3_ were recorded using a Bruker UltraShield Spectrometer (400 MHz) at room temperature. The ^1^H and ^13^C{^1^H} chemical shifts were referenced to the residual signals of the protons or carbons of the NMR solvents and are quoted in ppm, i.e., CD_3_OD at 4.79 and 49.00 ppm for ^1^H NMR and ^13^C{^1^H} spectra, respectively, and CDCl_3_ at 7.24 and 77.00 ppm for ^1^H and ^13^C{^1^H} spectra, respectively. The infrared spectrum was recorded using a Bruker Tensor 27 Spectrometer fitted with an ATP-IR probe and PerkinElmer FTIR Spectrum BX. Elemental analysis was performed using a Vario Elementar III Microcube CHNS Analyzer at the University of Johannesburg, South Africa.

### Synthesis of [2-(diphenylphosphino)ethyl]amine gold(I) chloride (1)

Title compound **1** was prepared according to a previous procedure (Jiang, et al., 2009) from [Au(tht)Cl], which was obtained by reacting H[AuCl_4_].3H_2_O with tetrahydrothiophene (tht) in ethanol. A dichloromethane solution of [Au(tht)Cl] (0.26 g, 0.87 mmol) was added to a solution of 2-(diphenylphosphino)ethanamine (**L1**) (0.20 g, 0.87 mmol) in dichloromethane (10 mL) and stirred for 3 h. The solvent was reduced to half, and hexane was added until a white precipitate was formed. The white solid obtained was filtered and dried *in vacuo*.

^1^H NMR (CDCl_3_, 400 MHz) σ_H_: 7.68 (q, 4H, J = 7.6 Hz, Ph_2_), 7.46 (q, 6H, J = 9.6 Hz, Ph_2_), 3.06 (m, 2H, CH_2_), 2.70 (q, 2H, J = 8.4 Hz, CH_2_), and 1.64 (s, 2H, NH_2_). ^13^C{^1^H} NMR (CDCl_3,_ 100 MHz**)** δ_C_: 133.2 (Ph), 133.1 (Ph), 129.5 (Ph), 129.3, 128.8, (Ph), 128.9 (Ph), 38.7 (CH_2_), and 32.3 (CH_2_); ^31^P{^1^H} NMR δ: 25.95 (-PPh). The elemental analysis showed calculated values for C_14_H_16_AuClNP as C, 36.42; H, 3.49; and N, 3.03, with found values of C, 36.35; H, 3.32; and N, 3.05.

### Synthesis of [3-(diphenylphosphino)propyl]amine gold(I) chloride (2)

Complex **2** was prepared using similar method discussed for complex **1** with the following reagents: 3-(diphenylphosphino) propanamine (0.44 g, 1.8 mmol) and Au(tht)Cl (0.58 g, 1.8 mmol).

^1^H NMR (CDCl_3_, 400 MHz): σ_H_ 7.52 (q, 2H, J = 7.6 Hz, Ph_2_), 7.44 (q, 3H, J = 7.6 Hz, Ph_2_), 2.82 (t, 2H, J = 7.6 Hz, CH_2_), 2.67 (q, 3H, J = 10 Hz, CH_2_), 1.77 (q, 3H, J = 10 Hz, CH_2_), and 1.56 (s, 2H, NH_2_); ^13^C{^1^H} NMR (CDCl_3,_ 100 MHz**)** δ_C_: 133.2 (Ph), 133.1 (Ph), 129.5 (Ph), 125.3, 129.3 (Ph), 128.9 (Ph), 42.5 (CH_2_), 29.1 (CH_2_), and 25.8 (CH_2_); ^31^P{^1^H} NMR δ: 34.01 (-PPh). The elemental analysis showed calculated values for C_15_H_18_AuClNP as C, 37.87; H, 3.81; and N, 2.91, with found values of: C, 37.12; H, 3.91; and N, 2.85.

### Synthesis of 2-gluconamido ethane-2-(diphenylphosphino)ethyl]amine gold(I) chloride (3)

Complex **3** was synthesized according to a previous method (Coetzee et al., 2007) with slight modifications. D-(+)-Gluconic acid δ-lactone (0.1 g, 0.56 mmol) was dissolved in methanol (30 mL) at 50°C. After the solution cooled down to room temperature, [2-(diphenylphosphino)ethyl]amine gold(I) chloride (**1**) (0.26 g, 0.56 mmol) was added. The mixture was then stirred overnight at room temperature, and the solution was then reduced to half, followed by the addition of hexane until a white precipitate formed. The white precipitate obtained was filtered and washed successively with hexane (3 × 10 mL) to yield a white solid of compound **3**. Yield: 0.31 g (86%). ^1^H NMR (CD_3_OD, 400 MHz) σ_H_: 7.65 (m, 4H, Ph), 7.43 (q, 6H, J = 7.2 Hz, Ph), 4.51 (s, 1H, OH), 4.06 (s, 1H, OH), 3.98 (s, 2H, OH), 3.65 (s, 1H, OH), 3.63 (s, 1H, H-2), 3.61 (m, 5H, H3-6), 3.40 (q, 2H, J = 6.8, C7), and 2.85 (s, 2H, br. C8); ^13^C{^1^H} NMR (CD_3_OD, 100 MHz) δ_C_: 179.1 (C=O), 133.1 (Ph), 132.3 (Ph), 131.9 (Ph), 129.5, 129.3 (Ph), 129.2 (Ph), 73.7 (C-2), 73.1 (C-3), 72.6 (C-4), 71.6 (C-5), 63.34 (C-6), 35.2 (CH_2_), and 35.1 (CH_2_); ^31^P{^1^H} NMR δ: 37.57 and 23.46 (PPh); FTIR (neat, cm^-1^): 3,269 (O-H), 2,917 (C-H), 1,647 (C=O), 1,527 (N-H), 1,434 (CH_2_ bend), 1,101 (O-C), 1,025 (C-N), and 739, 690 (Ph, C-H). The elemental analysis showed calculated values for C_20_H_26_AuClNO_6_P as C, 37.54; H, 4.10; and N, 2.91, with found values of C, 38.04; H, 4.49; and N, 2.50.

### Synthesis of [3-gluconamido ethane-3-(diphenylphosphino)propyl]amine gold(I) chloride (4)

Complex **4** was synthesized according to the method described for complex **3** with the following reagents: D-(+)-gluconic acid δ-lactone (0.06 g, 0.34 mmol) and [2-(diphenylphosphino)ethyl]amine gold(I) chloride (**1**) (0.15 g, 0.34 mmol). Yield = 0.19 g (86%).

^1^H NMR (CD_3_OD, 400 MHz) δ_H_: 7.72 (s, br, 4H, Ph_2_), 7.50 (s, br, 6H, Ph_2_), 4.14 (d, 1H, J = 2.8, H-3), 4.02 (s, 1H, H-2), 3.62 (m, 3H, H5-6), 3.52 (q, 1H, H-4), 2.78 (s, 2H, br, C7), 1.83 (s, 2H, br. C8), and 1.80 (s, br, 2H, C9); ^13^C{^1^H} NMR (CD_3_OD, 100 MHz) δ_C_: 178.3 (C=O), 137.1 (Ph), 136.1 (Ph), 135.9 (Ph), 133.4 (Ph), 78.0 (C-2), 76.8 (C-3), 75.5 (C-4), 74.6 (C-5), 67.2 (C-1), 29.3 (CH_2_), 28.4 (CH_2_), and 27.6 (CH_2_); ^31^P{^1^H} NMR (CD_3_OD) δ: 40.16 and 30.32 (PPh); FTIR (neat, cm^-1^): 3,291 (O-H), 2,925 (CH_2_), 1,644 (C=O), 1,534 (N-H), 1,435 (CH_2_ bend), 1,102–1077 (O-C), 1,025 (C-N), and 740, 691 (Ph, C-H). The elemental analysis showed calculated values for C_21_H_28_AuClNO_6_P as C, 38.58; H, 4.32; and N, 2.14, with found values of C, 38.18; H, 3.78; and N, 2.50.

### Synthesis of [acetylated-2-gluconamido ethyl (diphenylphosphino)] gold(I) chloride (5)

To a solution of complex **3** (0.12 g, 0.18 mmol) and acetic anhydride (0.10 mL, 1.10 mmol) (1:1 ratio to the hydroxyl group) in pyridine (2 mL), a catalytic amount of DMAP was added. The solution was stirred at room temperature for 18 h. The mixture was then diluted with dichloromethane (20 mL) and washed with 1M HCl_(aq)_ solution (5 × 30 mL). The organic layer was dried using anhydrous MgSO_4_, after which the solvent was pumped off using a rotary evaporator to obtain a white solid. Yield: 0.16 g (84%).

^1^H NMR (CDCl_3_, 400 MHz) δ_H_: 7.66 (q, 4H, J = 6.8 Hz, Ph_2_), 7.47 (m, 6H, Ph_2_), 6.67 (t, 1H, J = 6.0 Hz, NH), 5.58 (t, 1H, J = 4.8 Hz, H-3), 5.43 (t, 1H, J = 6, H-4), 5.19 (d, 1H, J = 4.8, H-2), 5.03 (q, 1H, J = 5.6, H-5), 4.31 (dd, 1H, J = 3.2 and 12.4 Hz, H-6a), 4.09 (dd, 1H, J = 5.6 and 12.4 Hz, H-6b), 3.46 (t, 2H, J = 7.2, CH_2_), 2.73 (m, 4H, C7-8), 2.18 (s, 3H, CH_3_), 2.08 (s, 3H, CH_3_), 2.04 (s, 3H, CH_3_), 2.03 (s, 3H, CH_3_), and 2.00 (s, 3H, CH_3_); ^13^C{^1^H} NMR (CDCl_3,_ 100 MHz) δ_C_: 170.7 (-OCOCH_3_), 169.9 (-OCOCH_3_), 169.7 (-OCOCH_3_), 167.0 (-OCOCH_3_), 164.5 (C-1), 133.3 (Ph), 133.2 (Ph), 132.3 (Ph), 129.5 (Ph), 129.4 (Ph), 71.8 (C-2), 71.2 (C-3), 69.5 (C-4), 68.9 (C-5), 61.7 (C-6), 36.0(-NHCH_2_-), 34.1 (-CH_2_P-), 20.7 (3×-OCOCH_3_), and 20.5 (2×-OCOCH_3_); ^31^P{^1^H} NMR δ: 23.81 (PPh); FTIR (neat, cm^-1^): 1,744 (C=O, ester), 1,677 (C=O), 1,525 (N-H), 1,436 (=CH_2_), 1,370 (-CH_3_), 1,211 (O-C), 1,044 (C-N), and 743, 693 (Ph). The elemental analysis showed calculated values for C_30_H_36_AuClN_2_O_11_P as C, 42.39; H, 4.27; and N, 1.65, with found values of C, 42.39; H, 4.60; and N, 2.07.

### Synthesis of [acetylated-3-gluconamido propyl (diphenylphosphino)] gold(I) chloride (6)

To a solution of complex **4** (0.10 g, 0.15 mmol) and acetic anhydride (0.08 mL, 0.75 mmol) (1:1 ratio to the hydroxyl group) in pyridine (2 mL), a catalytic amount of DMAP was added. The solution was stirred at room temperature for 18 h. The mixture was then diluted with dichloromethane (20 mL) and washed with 1M HCl_(aq)_ solution (5 × 30 mL). The organic layer was dried using anhydrous MgSO_4_, after which the solvent was pumped off using a rotary evaporator to obtain a white solid. Yield: 0.11 g (85%).

^1^H NMR (CDCl_3_, 400 MHz) δ_H_: 7.69 (d, 4H, J = 6.8 Hz, Ph_2_), 7.47 (m, 6H, Ph_2_), 5.68 (t, 1H, J = 4.8 Hz, H-3), 5.46 (t, 1H, J = 6, H-4), 5.28 (d, 1H, J = 4.8, H-2), 5.05 (q, 1H, J = 6.0, H-5), 4.31 (dd, 1H, J = 3.6 and 12.0 Hz, H-6a), 4.12 (dd, 1H, J = 5.6 and 11.6 Hz, H-6b), 3.41 (d, 2H, J = 5.6, C7), 2.24 (s, 3H, CH_3_), 2.08 (s, 3H, CH_3_), 2.04 (s, 3H, CH_3_), 2.01 (s, 3H, CH_3_), 1.98 (s, 3H, CH_3_), 1.823 (q, 2H, C8), and 1.65 (s, 2H, C9); ^13^C{^1^H} NMR (CDCl_3,_ 100 MHz) δ_C_: 170.7 (-OCOCH_3_), 170.2 (-OCOCH_3_), 169.9 (-OCOCH_3_), 169.8 (-OCOCH_3_), 166.9 (C-1), 133.3 (Ph), 133.2 (Ph), 131.8 (Ph), 129.3 (Ph), 72.3 (C-2), 69.6 (C-3), 69.0 (C-4), 68.7 (C-5), 61.7 (C-6), 39.2 (C7), 31.6 (C8), 24.9 (C9), 21.0 (2×-OCOCH_3_), 20.8 (OCOCH_3_), 20.7 (OCOCH_3_), and 20.5 (OCOCH_3_); ^31^P{^1^H} NMR: 33.83 (PPh); FTIR (neat, cm^-1^): 2,923 (C-H), 1,742 (C=O, ester), 1,672 (C=O), 1,531 (N-H), 1,435 (CH_2_), 1,369 (CH_3_), 1,209 (O-C), 1,042 (C-N), and 734, 692 (Ph). The elemental analysis showed calculated values for C_31_H_38_AuClNO_11_P as C, 43.09; H, 4.43; and N, 1.62, with found values of C, 44.39; H, 4.42; and N, 1.77.

### Synthesis of [2-(diphenylphosphino)ethyl]amine acetyl-2-gluconamido ethane thiolate gold(I) complex (7)

Complex **1** (0.2 g, 0.43 mmol) and acetylated 2-gluconamido ethane thiol (0.2 g, 0.43 mmol, 1 equivalent) were dissolved in 20 mL of dichloromethane, and 0.06 mL of triethylamine was added. The reaction mixture was stirred overnight at ambient temperature. A white precipitate formed upon the addition of hexane, which was filtered by suction filtration and dried *in vacuo* to yield a white solid. Yield = 0.32 g (84%).

^1^H NMR (CDCl_3_, 400 MHz) σ_H_: 7.46 (t, 4H, J = 8.4, Ph), 7.42 (q, 6H, J = 4.8, Ph), 7.11 (s, 1H, NH), 5.65 (t, 1H, J = 4.8, H-3), 5.47 (t, 1H, J = 6, H-4), 5.35 (t, 1H, J = 4.4, H-2), 5.03 (q, 1H, J = 5.6, H-5), 4.30 (dd, 1H, J = 4.0 and 12.0 Hz, H-6a), 4.10 (dd, 1H, J = 6.0 and 14 Hz, H-6b), 3.46 (t, 2H, J = 7.2, C7), 3.15 (s, 4H, br. C8-9), 2.96 (s, 2H, C10), 2.18 (s, 3H, CH_3_), 2.07 (s, 3H, CH_3_), 2.05 (s, 3H, CH_3_), 2.03 (s, 3H, CH_3_), 2.00 (s, 3H, CH_3_), and 1.38 (q, 3H, J = 7.6, NH_2_); ^31^P{^1^H} NMR: 29.25 (PPh); ^13^C{^1^H} NMR (CDCl_3,_ 100 MHz) δ_C_: 170.6 (-OCOCH_3_), 169.8 (-OCOCH_3_), 169.4 (-OCOCH_3_), 166.5 (-OCOCH_3_), 166.1 (C-1), 133.3 (Ph), 132.0 (Ph), 129.4 (Ph), 72.1 (C-2), 69.7 (C-3), 69.2 (C-4), 68.7 (C-5), 61.6 (C-6), 44.8 (-NHCH_2_-), 31.6 (-CH_2_S-), 32.7 (NH_2_CH_2_-), 20.9 (-OCOCH_3_), 20.8 (2×-OCOCH_3_), 20.7 (-OCOCH_3_), 20.5 (-OCOCH_3_), and 14.1 (-PCH_2_-); FTIR (neat, cm^-1^): 1,745 (C=O, ester), 1,673 (C=O), 1,526 (N-H), 1,436 (=CH_2_), 1,370 (CH_3_), 1,211 (O-C), 1,045 (C-N), and 958, 742, 693 (Ph). The elemental analysis showed calculated values for C_32_H_42_AuN_2_O_11_PS as C, 43.15; H, 4.75; N, 3.15; and S, 3.60, with found values of C, 42.69; H, 4.74; and N, 2.93.

### Synthesis of [3-(diphenylphosphino)propyl]amine acetyl-2-gluconamido ethane thiolate gold(I) complex (8)

Complex **2** (0.20 g, 0.42 mmol) and acetylated 2-gluconamido ethane thiol (0.20 g, 0.43 mmol, 1 equivalent) were dissolved in 20 mL of dichloromethane, and 0.06 mL of triethylamine was added. The reaction mixture was stirred overnight at ambient temperature. A white precipitate formed upon the addition of hexane, which was filtered by suction filtration and dried *in vacuo* to yield a white solid. Yield = 0.33 g (87%).

^1^H NMR (CDCl_3_, 400 MHz) σ_H_: 7.54 (s, br, 4H, Ph), 7.41 (q, br, 6H, Ph), 7.03 (s, 1H, NH), 5.66 (t, 1H, J = 4.8 Hz, H-3), 5.47 (t, 1H, J = 5.6 Hz, H-4), 5.35 (d, 1H, J = 3.6 Hz, H-2), 5.04 (q, 1H, J = 4.0 Hz, H-5), 4.30 (dd, 1H, J = 4.0 and 12.0 Hz, H-6a), 4.11 (dd, 1H, J = 5.6 and 12.4 Hz, H-6b), 3.50 (s, 2H, C7), 3.05 (s, 2H, br. C8), 2.92 (s, br, 2H, C9), 2.53 (s, 2H, C11), 2.20 (s, 3H, CH_3_), 2.07 (s, 6H, CH_3_), 2.04 (s, 3H, CH_3_), 2.02 (s, 3H, CH_3_), 1.79 (s, 2H, C10), and 1.26 (t, 2H, J = 8.0, NH_2_); ^31^P{^1^H} NMR: 34.67 (PPh); ^13^C{^1^H} NMR (CDCl_3,_ 100 MHz) δ_C_: 170.6 (-OCOCH_3_), 169.9 (-OCOCH_3_), 169.8 (-OCOCH_3_), 169.7 (-OCOCH_3_), 169.3 (-OCOCH_3_), 166.1 (C-1), 131.3 (Ph), 129.1 (Ph), 72.1 (C-2), 69.6 (C-3), 69.0 (C-4), 68.7 (C-5), 61.5 (C-6), 52.8 (-NHCH_2_-), 41.86 (-CH_2_S-), 31.5 (NH_2_CH_2_-), 20.8 (3×-OCOCH_3_), 20.7 (2×-OCOCH_3_), 20.7 (-OCOCH_3_), 14.1 (-PCH_2_-), and 7.91 (-CH_2_-); FTIR (neat, cm^-1^): 1,744 (C=O, ester), 1,670 (C=O), 1,525 (N-H), 1,435 (=CH_2_), 1,370 (CH_3_), 1,211 (O-C), 1,044 (C-N), and 958, 743, 694 (Ph). The elemental analysis showed calculated values for C_33_H_44_AuN_2_O_11_PS as C, 43.81; H, 4.90; N, 3.10; and S, 3.37, with found values of C, 42.69; H, 5.18; and N, 3.13.

### Synthesis of bimetallic complexes

#### Dichloro{bis[2-(diphenylphosphino)ethyl]amino gold(I) acetyl-2-gluconamido ethane thiolate} platinum(II) chloride (9)

To a solution of complex **7** (0.05 g, 0.056 mmol) in dichloromethane (10 mL), [Pt(COD)Cl_2_)] (0.042 g, 0.11 mmol) was added and stirred for 18 h. The solution was filtered, and hexane was added until a white precipitate formed. It was then filtered and dried *in vacuo*. Yield = 0.081 g (73.3%).

^1^H NMR (CDCl_3_, 400 MHz) σ_H_: 8.02 (s, 1H, Ph), 7.73 (d, 3H, J = Hz, Ph), 7.49 (t, 3H, J = Hz, Ph), 7.40 (s, 3H, Ph), 5.98 (s, 1H, H-3), 5.64 (d, 1H, J = 5.2 Hz, H-2), 5.39 (t, 1H, J = 3.2 Hz, H-5), 5.04 (s, 1H, H-4), 4.28 (d, 1H, J = 3.2 Hz, H-6a), 4.11 (d, 1H, J = 6.2 Hz, H-6b), 3.46 (d, 2H, J = 7.6 Hz, CH_2_), 3.31 (d, 2H, J = 6.8 Hz, CH_2_), 3.04 (s, 2H, J = 7.6 Hz, CH_2_), 2.66 (s, 2H, CH_2_), 2.24 (s, 3H, CH_3_), 2.07 (s, 3H, CH_3_), 2.03 (s, 3H, CH_3_), 1.98 (s, 3H, CH_3_), and 1.72 (s, 3H, CH_3_). ^31^P{^1^H} NMR: 33.02, 23.97 (PPh); ^13^C{^1^H} NMR (CDCl_3,_ 100 MHz**)** δ_C_: 172.5 (-OCOCH_3_), 170.1 (-OCOCH_3_), 169.9 (-OCOCH_3_), 167.8 (-OCOCH_3_), 166.5 (C-1), 158.5 (PyC13), 152.5 (PyC12), 141.6 (PyC11), 140.2 (PyC10), 136.7 (PyC9), 134.0 (Ph), 129.7 (Ph), 129.6 (Ph), 127.1 (Ph), 71.0 (C-2), 70.1 (C-3), 69.6 (C-4), 69.1 (C-5), 61.6 (C-6), 45.2(C7), 45.0 (C8), 21.1 (OCOCH_3_), 20.8 (-OCOCH_3_), 20.7 (2×-OCOCH_3_), and 20.6 (-OCOCH_3_); FTIR (neat, cm^-1^): 1,747 (C=O, ester), 1,660 (C=O), 1,528 (N-H), 1,433 (CH_2_), 1,214 (CH_3_), 1,103 (O-C), 1,046 (C-N), 956 (Ph), 742 (P-C), and 695 (C-S). The elemental analysis showed calculated values for C_64_H_82_Au_2_Cl_2_N_4_O_22_P_2_PtS_2_ as C, 37.58; H, 4.04; N, 2.74; and S, 3.13, with found values of C, 38.11; H, 4.90; and N, 2.87. HR-MS (ESI) analysis showed a calculated *m/z* [M-Cl]^+^ of 1,973.3316, while the found value was 1,973.2434.

#### Dichloro{[2-(diphenylphosphino)ethyl]amino gold(I) acetyl-2-gluconamido ethane thiolate}palladium(II) chloride (10)

To a solution of complex **7** (0.05 g, 0.0 mmol) in dichloromethane (10 mL), [Pd(CNMe)_2_Cl_2_)] (14 mg, 0.06 mmol) was added and stirred for 18 h. The solution was filtered, and hexane was added until a yellow precipitate formed. It was then filtered and dried *in vacuo*. Yield = 0.0 g (%). ^1^H NMR (CDCl_3_, 400 MHz) σ_H_: 7.85 (t, 1H, J = 5.4 Hz, Ph), 7.71 (q, 4H, J = 5.2 Hz, Ph), 7.47 (q, 3H, J = 8.0 Hz, Ph), 7.39 (s, 3H, Ph), 5.64 (t, 1H, J = 4.8 Hz, H-3), 5.43 (t, 1H, J = 5.2 Hz, H-2), 5.31 (t, 1H, J = 4.0 Hz, H-5), 5.04 (s, 1H, H-4), 4.29 (d, 1H, J = 3.2 Hz, H-6a), 4.13 (q, 1H, J = 5.6 Hz, H-6b), 3.44 (d, 2H, J = 7.6 Hz, CH_2_), 3.27 (t, 2H, J = 6.8 Hz, CH_2_), 2.92 (t, 2H, J = 8.0 Hz, CH_2_), 2.76 (s, 2H, CH_2_), 2.18 (s, 3H, CH_3_), 2.08 (s, 3H, CH_3_), 2.07 (s, 3H, CH_3_), 2.06 (s, 3H, CH_3_), and 2.00 (s, 3H, CH_3_). ^31^P{^1^H} NMR: 54.87, 23.95 (PPh); ^13^C{^1^H} NMR (CDCl_3,_ 100 MHz) δ_C_: 170.8 (-OCOCH_3_), 170.7 (-OCOCH_3_), 170.9 (-OCOCH_3_), 170.8 (-OCOCH_3_), 169.5 (-OCOCH_3_), 133.5 (Ph), 132.0 (Ph), 129.5 (Ph), 129.4 (Ph), 129.3 (Ph), 71.0 (C-2), 69.7 (C-3), 69.0 (C-4), 62.9 (C-5), 61.5 (C-6), 55.0 (CH_2_), 53.1 (CH_2_), 52.0 (CH_2_), 21.5 (OCOCH_3_), 20.7 (-OCOCH_3_), 20.7 (-OCOCH_3_), 20.4 (-OCOCH_3_), and 20.2 (-OCOCH_3_); FTIR (neat, cm^-1^): 1,747 (C=O, ester), 1,665 (C=O), 1,528 (N-H), 1,433 (CH_2_), 1,369 (CH_3_), 1,214 (O-C), 1,043 (C-N), 959 (Ph), 742 (P-C), and 692 (C-S). The elemental analysis showed calculated values for C_32_H_40_AuCl_2_N_2_O_11_PPdS.CH_2_Cl_2_ as C, 34.44; H, 3.68; and N, 2.43, with found values of C, 33.93; H, 4.39; and N, 2.74. HR-MS (ESI) analysis showed a calculated *m/z* [M]^+^ of 1,064.0168, while the found value was 1,064.1804.

#### Dichloro{bis[2-(diphenylphosphino)propyl]amino gold(I) acetyl-2-gluconamido ethane thiolate}palladium(II) chloride(11)

To a solution of complex **8** (100 mg, 0.11 mmol) in dichloromethane (20 mL), [Pd(CNMe)_2_Cl_2_)] (14 mg, 0.06 mmol) was added and stirred for 18 h. The solution was filtered and reduced to about 5 mL. Then, 5 mL hexane was added until a yellow precipitate was formed. It was then filtered and dried *in vacuo*. Yield = 0.07 g (64%). ^1^H NMR (CDCl_3_, 400 MHz) σ_H_: 7.64 (d, 4H, J = 8.8 Hz, Ph), 7.48 (s, 3H, J = 8.0 Hz, Ph), 7.38 (s, 3H, Ph), 5.64 (s, 1H, H-3), 5.42 (s, 1H, H-2), 5.31 (s, 1H, H-5), 5.03 (s, 1H, H-4), 4.29 (d, 1H, J = 3.2 Hz, H-6a), 4.11 (t, 1H, J = 6.0 Hz, H-6b), 3.43 (q, 2H, J = 10.0 Hz, CH_2_), 3.26 (d, 2H, J = 6.8 Hz, CH_2_), 2.75 (s, 2H, CH_2_), 2.65 (s, 2H, CH_2_), 2.19 (s, 3H, CH_3_), 2.08 (s, 3H, CH_3_), 2.06 (s, 3H, CH_3_), 2.04 (s, 3H, CH_3_), 2.02 (s, 3H, CH_3_), and 1.33 (t, 2H, J = 6.8 Hz, CH_2_). ^31^P{^1^H} NMR: 54.87, 23.95 (PPh); ^13^C{^1^H} NMR (CDCl_3,_ 100 MHz) δ_C_: 170.6 (-OCOCH_3_), 169.9 (-OCOCH_3_), 169.8 (-OCOCH_3_), 168.5 (-OCOCH_3_), 168.2 (-OCOCH_3_), 133.5 (Ph), 133.3 (Ph), 132.0 (Ph), 129.5 (Ph), 129.4 (Ph), 71.8 (C-2), 69.8 (C-3), 69.0 (C-4), 61.6 (C-5), 61.5 (C-6), 53.3 (CH_2_), 41.6 (CH_2_), 37.8 (CH_2_), 20.8 (OCOCH_3_), 20.7 (-OCOCH_3_), 20.7 (-OCOCH_3_), 20.4 (-OCOCH_3_), and 20.2 (-OCOCH_3_); FTIR (neat, cm^-1^): 1,747 (C=O, ester), 1,663 (C=O), 1,531 (N-H), 1,436 (CH_2_), 1,370 (CH_3_), 1,212 (O-C), 1,046 (C-N), 959 (Ph), 745 (P-C), and 692 (C-S). The elemental analysis showed calculated values for C_64_H_82_Au_2_Cl_2_N_4_O_22_P_2_PdS_2_ as C, 39.29; H, 4.22; N, 2.86; and S, 3.28, with found values of C, 39.82; H, 3.90; and N, 2.33.

### Computational methodology

#### Molecular docking

**FIGURE 2:**
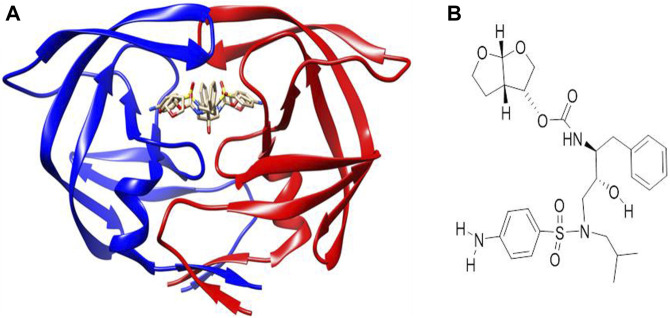
**(A)** Co-crystallized X-ray crystallographic structure of HIV protease bound to darunavir (PDB code 4LL3). **(B)** Structure of darunavir.

AutoDock Vina (Trott and Olson, 2010) was used for the molecular docking of the synthesized mono- and bimetallic glyco diphenylphosphino gold(I), palladium(II), and platinum(II) thiolate complexes, and darunavir (an antiretroviral drug used to treat and prevent HIV/AIDS) as a control drug (Figure 2B) to the inhibitor-binding domain of HIV protease was analyzed using UCSF Chimera (Pettersen et al., 2004; Kožíšek et al., 2014). While the 3D structure of HIV protease was retrieved from the Protein Data Bank [https://www.rcsb.org] with code 4LL3 (Kožíšek et al., 2014), the structures of the metal complexes were drawn using MarvinSketch (ChemAxon, 2013) and subsequently optimized and converted to 3D using Avogadro (Hanwell et al., 2012). The 3D structure of HIV protease was prepared by removing co-crystallized water molecules, non-polar hydrogens, and other non-standard residues using UCSF Chimera. The binding site for molecular docking was defined using a co-crystallized known inhibitor of HIV protease, darunavir (Figure 2). To map out the binding site, a grid box was used with dimensions centered at x = −8.58, y = 16.05, and z = 27.27 and size: x = 11.06, y = 6.35, and z = 16.01. To prepare for molecular docking, hydrogen and Gasteiger charges were added to the metal complexes, after which each compound was subsequently set as flexible, while the receptor was positioned in a rigid conformation using UCSF Chimera. The default exhaustiveness of 8 for AutoDock Vina was used to allow for a thorough conformation search and optimization. Compounds that showed the top three docking scores (mostly negative) were selected for further analysis via molecular dynamics (MD).

#### Molecular dynamics simulation of protein–ligand complexes

Molecular dynamics was performed using the GPU version of AMBER 18 with an incorporated PMEMD module (Le Grand et al., 2013) following standard protocols as elaborated in several computational studies (Ramharack et al., 2023; Agoni et al., 2018a; Agoni et al., 2018b; Olotu et al., 2019; Agoni et al., 2022). In all, four complexes generated from molecular docking were prepared for molecular dynamics simulation. The corresponding unbound (apo) form of the HIV protease was also prepared for simulation.

The compounds were parameterized using the ANTECHAMBER module, where atomic partial charges (AM1BCC) of Gaff, using the bcc charge scheme, were added (Salomon-Ferrer et al., 2013; Maier et al., 2015). For the gold atoms, parameters were derived using a combination of quantum mechanical calculations and established parameters from literature specific to gold (Mohammadnejad et al., 2015). Partial charges for the gold-containing ligands were assigned using the restrained electrostatic potential (RESP) method, following geometry optimization and electrostatic potential calculations at the HF/6-31G* level for non-metal atoms and a suitable level of theory for gold atoms (Schauperl et al. (2020). Bonding parameters for gold–ligand interactions were derived from quantum mechanical calculations and validated against experimental data when available. Non-bonding parameters, including van der Waals (vdW) interactions, were adjusted to accurately reflect the behavior of gold in a biological context.

HIV protease was also parameterized using the Amber FF14SB force field (Salomon-Ferrer et al., 2013; Maier et al., 2015). Using the LEAP module, hydrogen atoms were also added, while the entire system was neutralized by adding counter ions (Na^+^ and Cl^−^), followed by a subsequent generation of ligand, protein, and complex topologies, as well as parameter files for each of the molecules. The systems were precisely solvated with water molecules using the TIP3P orthorhombic box with a size of 12 Å (Case et al., 2005). The solvated complexes were minimized initially for 2,000 minimization steps, applying a restraint potential of 500 kcal/mol, and then fully minimized for another 1,000 steps of the steepest descent without restraint. This was followed by the gradual heating of the systems from 0 K to 300 K for 50 ps, after which they were equilibrated for 500 ps, while the temperature and pressure were kept constant at 300 K and 1 bar, respectively (Berendsen et al., 1984). This was followed by MD production runs of 300 ns for each system, during which the SHAKE algorithm (Kräutler et al., 2001) was used to constrain all atomic hydrogen bonds. The MD simulation was initiated using a time step of 1 fs and coordinates saved at 1-ps intervals, followed by the subsequent analysis of trajectories using the integrated PTRJ and CPPTRAJ module (Roe and Cheatham III, 2013). The complexes and data plots were visualized using the graphical user interface of UCSF Chimera and Microcal Origin analytical software (Seifert, 2014).

### Protonation state assignment

The accurate determination of protonation states for key residues in the active site is crucial for the fidelity of MD simulations and the design of effective protease inhibitors. HIV-1 protease contains two critical aspartate residues (Asp25 and Asp125) in its active site, which play a vital role in its catalytic mechanism (Adachi et al., 2009; Ghosh et al., 2016). Based on well-established practices and previous studies, these aspartate residues were modeled in their deprotonated forms in our simulations (Privat et al., 2020). This decision is supported by the typical pKa values of aspartate residues in enzymatic active sites, which suggest that they remain deprotonated under physiological pH conditions to act as nucleophiles in the catalytic process (Hofer et al., 2020).

#### Post-molecular dynamic simulation analysis

After a 300-ns MD simulation run, the coordinates of all the simulated systems were saved at 1-ps intervals, followed by the subsequent analysis of trajectories using the integrated PTRJ and CPPTRAJ module. The root mean square deviation (RMSD), root mean square fluctuation (RMSF), radius of gyration (RoG), and thermodynamic calculations were calculated and plotted for each of the resultant trajectories. The complexes and data plots were visualized using the graphical user interface of Discovery Studio (BIOVIA Discovery Studio, 2016) and Microcal Origin analytical software (Dassault Systèmes, 2016).

#### Thermodynamic calculations

The molecular mechanics/generalized born surface area (MM/GBSA) method was used to calculate the binding free energy of the protein–ligand complexes (Miller et al., 2012; Genheden and Ryde, 2015). The binding free energy **(ΔG**_**bind**_) of the protein–ligand complex was calculated as follows (Eqs 1–5):

$$\Delta G_{\text{bind}}=G_{\text{complex}}-G_{\text{receptor}}+G_{\text{ligand}},$$ (1)

$$\Delta G_{\text{bind}}=\Delta G_{\text{gas}}+\Delta G_{\text{sol}}-T\Delta S,$$ (2)

where $\Delta G_{bind}$ is the summation of the gas phase and solvation energy terms minus the entropy (TΔS) term.

$$\Delta E_{\text{gas}}=\Delta E_{\mathrm{int}}+\Delta E_{\text{vdw}}+\Delta E_{\text{elec}}.$$ (3)

$\Delta E_{\text{gas}}$ is the sum of the Amber force field internal energy terms $\Delta E_{\mathrm{int}}$ (bond, angle, and torsion), the covalent van der Waals ( $\Delta E_{\text{vdw}}$ ), and the non-bonded electrostatic energy component ( $\Delta E_{\text{elec}}$ ). The solvation energy is calculated as follows:

$$G_{\text{sol}}=G_{\text{GB}}+G_{\text{non}-\text{polar}},$$ (4)

$$G_{\text{non}\_\text{polar}}=\gamma \text{SASA}+b.$$ (5)

The polar solvation contribution is represented as $G_{\text{GB}}$ , and $G_{\text{non}\_\text{polar}}$ is the non-polar solvation contribution and is calculated from the solvent-assessable surface area (SASA), obtained using a water probe radius of 1.4 A°. The surface tension constant (c) was set to 0.0072 kcal/mol, and b was set to 0 kcal/mol.

Although MM/GBSA methods are widely used for estimating binding free energies in molecular dynamics simulations, it is important to acknowledge their limitations, particularly when applied to metal-containing systems. One major limitation is the accuracy of solvation energy calculations for metal ions and metal–ligand complexes, which may not be adequately captured by continuum solvent models (Rastelli et al., 2010; Kongsted et al., 2009). Several studies have highlighted the challenges in accurately describing the solvation behavior of metal ions and metal-containing compounds using MM/GBSA methods. For instance, the non-polar solvation term in MM/GBSA calculations relies on the SASA, which may not appropriately account for the complex solvation environments of metal ions (Wang et al., 2020). Additionally, the treatment of metal–ligand interactions, including electrostatic and van der Waals interactions, may require specialized parameterization and consideration of metal-specific effects, which are not fully addressed in standard MM/GBSA approaches (Genheden and Ryde, 2015).

## Results and discussion

**SCHEME 1:**
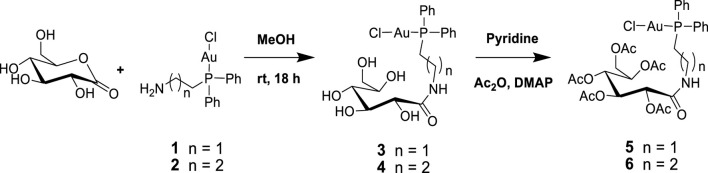
Synthesis of [2-gluconamido ethane *n*-(diphenylphosphino)alkyl]amine gold(I) complexes.

**SCHEME 2:**
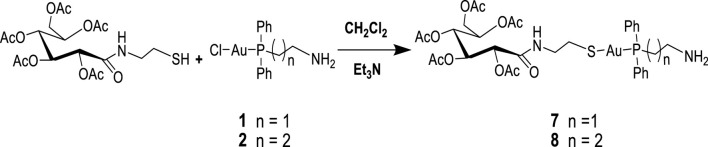
Synthesis of [diphenylphosphino)alkyl]amine acetylated 2-gluconamido alkane thiolate gold(I) complexes.

**SCHEME 3:**
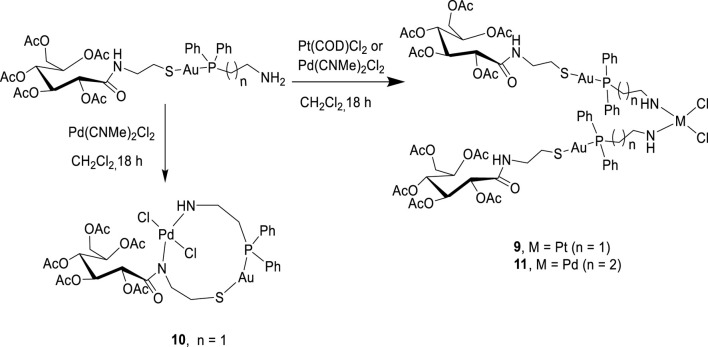
Synthesis of bimetallic complexes **9**–**11**.

Next, 2-diphenylphosphine amino gold chloride and 2-pyridyl diphenylphosphine gold(I) chloride were prepared from the appropriate amount of [(tht)AuCl] (tht = tetrahydrothiophene) (Jiang et al., 2009) by ligand substitution. Subsequent treatment with deprotonated thiocarbohydrate in dichloromethane, according to a procedure reported previously (Fillat et al., 2011), yielded four novel neutral products (**7, 8, 11,** and **12**), as shown in Schemes 2, 3. The [diphenylphosphinoalkyl]amine gold(I) chloride was treated with D-(+)-gluconic acid δ-lactone via the ring opening of D-(+) gluconic acid δ-lactone, leading to the formation of complexes **3** and **4** (Scheme 1)[1]. Further acetylation of **3** and **4** yielded complexes **5** and **6.**

All the new products are soluble in more polar and protic organic solvents, show good stability at room temperature, and are stable in air. Their stability and good solubility make them even more appealing candidates for biological screening since the stability of such compounds in solution is a vital consideration for biological evaluation. The complexes were characterized using spectroscopic techniques and microanalysis. The ^31^P{^1^H} NMR spectra of complexes **3** and **4** contain magnetically inequivalent phosphorus atoms, resulting in two peaks in each case, which resonated at 37.57 and 23.46 ppm for complex **3** and 40.16 and 30.32 ppm for **4,** compared with the precursors **1** and **2** at 25.95 and 34.01 ppm, respectively. This is attributed to the cis and trans analogs of the products. Acetylated complexes of **3** and **4** were also obtained from a reaction of **1** or **2** and the acetylated gluconamido ethyl thiol ligand and isolated as an off-white solid of **5** or **6**. The ^31^P{^1^H} NMR spectra of complexes **5** and **6** displayed a single peak in each case, which resonated upfield at 23.81 and 33.83 ppm, respectively, compared to the precursors. The phenyl carbon atoms display well-resolved signals in their ^13^C NMR spectra.

In order to study the structural activity of these compounds, modified congeners of **5** and **6** were synthesized by reacting 2-gluconamido ethyl thiol with compounds **1** or **2** in the presence of Et_3_N to yield complexes **7** or **8**, according to Scheme 2. The ^1^H and ^13^C{^1^H} NMR spectra showed the expected resonances. For example, the ^31^P{^1^H} NMR spectra of complexes **7** and **8** were measured at 29.25 and 34.67 ppm, respectively, which resonated downfield after the conjugation of acetylated 2-gluconamido ethane thiol with compounds **1** and **2**. The micro-analysis results of complexes **3**–**8** were in agreement with the calculated values, which confirmed the complexation and purity of these complexes.

Bimetallic complexes **9**–**11** were also readily prepared according to Scheme 3 from a reaction of [2-(diphenylphosphino)ethyl]amine acetyl-2-gluconamido ethane thiolate gold(I) complex (**7**) and [3-(diphenylphosphino)propyl]amine acetyl-2-gluconamido ethane thiolate gold(I) complex (**8**) with bis(benzonitrile) palladium(II) chloride [Cl_2_Pd(CNMe)_2_] or dichloro(1,5-cyclooctadiene) platinum(II) [Pt(COD)Cl_2_] (Scheme 3). The complexes were isolated in moderate yields and fully characterized by NMR, FTIR, and mass spectroscopy, as well as micro-analysis.

Complexes **9** and **11** were identified as bis-chelated dichloro{bis[2-(diphenylphosphino)ethyl]amino gold(I) acetyl-2-gluconamido ethane thiolate} platinum (II) and dichloro{bis[2-(diphenylphosphino)propyl]amino gold(I) acetyl-2-gluconamido ethane thiolate} palladium(II) complexes, respectively. In another development, a reaction of complex **7** with [Cl_2_Pd(CNMe)_2_] yielded chelated Pd(II) complex **10** having two nitrogen atoms (in the one carbohydrate thiolate amine moiety), one sulfur atom, and two chloride atoms coordinated to the Pd center. The high-resolution mass spectrum of **10** showed molecular ions (m/z = 1,062.1758) corresponding to the dichloro{[2-(diphenylphosphino)ethyl]amino gold(I) acetyl-2-gluconamido ethane thiolate} palladium(II) complex. This finding was also confirmed by micro-analysis.

### *In silico* exploration of the anti-HIV potential of the mono- and bimetallic glyco diphenylphosphino gold(I), palladium(II), and platinum(II) thiolate compounds

#### Molecular docking

To further establish the therapeutic potential of the mono and bimetallic glyco diphenylphosphino gold(I), palladium (II), and platinum (II) thiolate compounds synthesized, we explored their binding potential against HIV protease using molecular modeling techniques. This was premised on the previously reported inhibitory potential of gold(I) phosphine compounds against HIV-1 RT and HIV-1 PR by Fonteh and Meyer (2009). Molecular docking was performed, allowing for establishing the best binding poses of the compounds, which were subsequently scored for the ranking. The ligand-binding site of HIV protease was defined using its co-crystallized, known inhibitor. Docking scores that corresponded with compounds with the best pose were recorded and are given in Table 1. As shown, compounds **1, 2, 3, 4, 9**, **10**, and **11** showed higher binding energy scores than the standard inhibitor darunavir, suggesting the potential favorable inhibition of the enzyme. The top three potential inhibitors (compounds **2**, **3**, and **4**) were selected for further modeling to provide detailed insights into the potential binding of these compounds to HIV protease.

**TABLE 1:** Docking scores of the synthesized metal complexes toward HIV protease.

Compounds	Docking scores (kcal/mol)
1	−8.7
2	−9.1
3	−9.4
4	−9.9
5	−7.8
6	−7.4
7	−6.3
8	−7.1
9	−8.1
10	−7.9
11	−8.3
Darunavir (control)	−7.8

### Elucidating the structural binding mechanism of metal complexes relative to darunavir

The binding of darunavir to HIV protease for its inhibition is characterized by strong interactions between the 3(R),3a(S),6a(R)-bis-THF moiety of darunavir and the main-chain atoms of aspartates 29 and 30 (King et al., 2004; Ghosh et al., 2006). These interactions account for the more favorable binding enthalpy of darunavir to HIV protease than that of amprenavir, a chemically related inhibitor broadly used in antiviral therapy. Darunavir was therefore used as a control to assess the potential binding mechanism of compounds **2, 3**, and **4** toward HIV protease. Molecular docking allowed for the prediction of the binding modes of compounds within the inhibitor-binding site of HIV protease while also revealing the specific interactions that could inform their possible binding mechanism (Prieto-Martínez et al., 2018; Lin et al., 2020).

**FIGURE 3:**
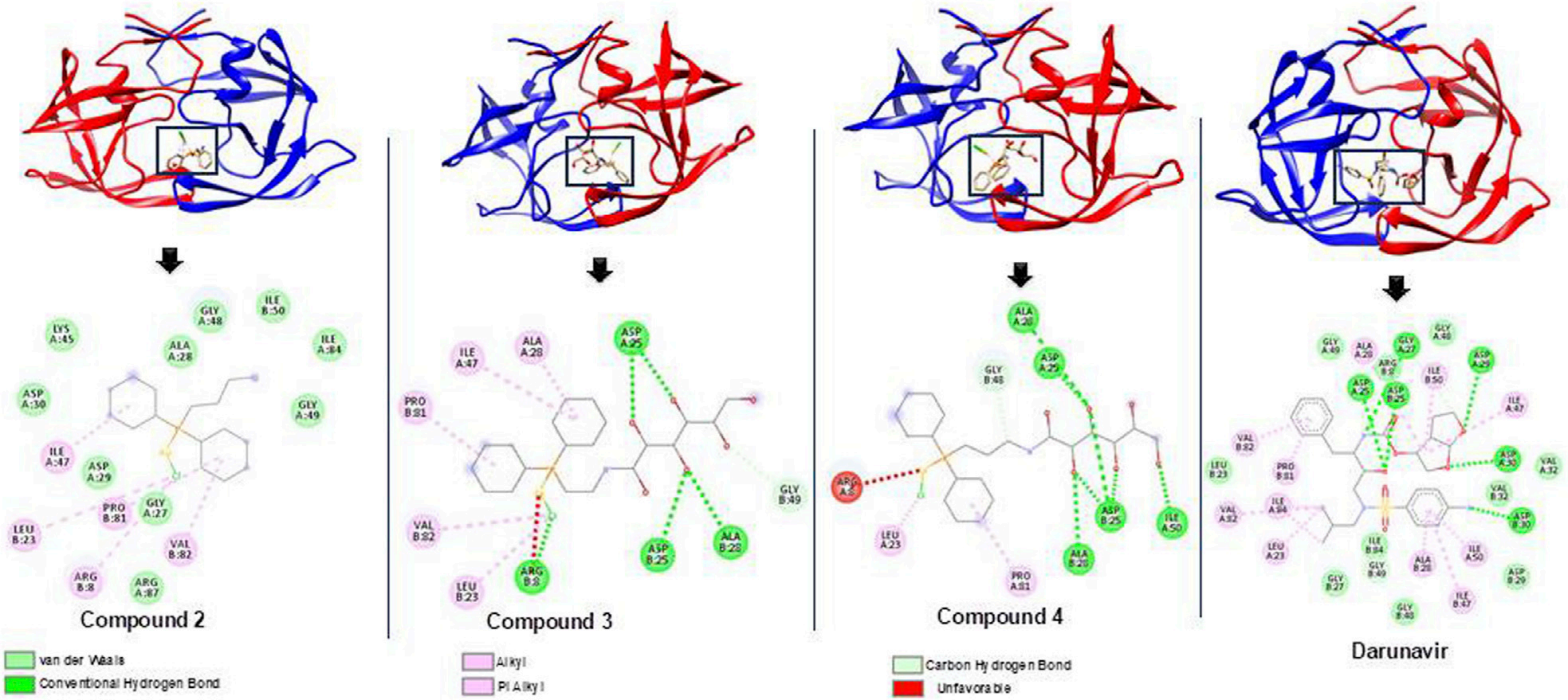
Bound conformations and corresponding protein–ligand interaction dynamics of metal complexes, compounds **2**, **3**, **and 4**, and the known HIV protease inhibitor, darunavir.

As shown in Table 1, docking scores of −9.1, −9.4, and −9.9 kcal/mol were calculated for compounds **2**, **3**, and **4**, respectively. As a control, darunavir was redocked into its binding pocket, showing a docking score of −7.8 kcal/mol. Redocking of darunavir also allowed for an assessment of the reliability and validation of the docking protocol used. The docking scores calculated represented the best-bound conformations and provided insights into the likely binding affinities of the compounds (Dar and Mir, 2017; Prieto-Martínez et al., 2018). The compounds exhibited relatively higher docking scores than darunavir. The strong interactions of darunavir with ASP29 and ASP30, as previously reported, were highlighted, characterized by conventional hydrogen bonds and van der Waals interactions (Figure 3). The binding of darunavir also included conventional hydrogen interactions with ASP25 and GLY27. The binding of compound **4** was characterized by conventional hydrogen bonds with ASP25, ALA28, and ILE50 in several π–alkyl and alkyl interactions with other binding pocket residues, notably pi–alkyl interactions between CL and LEU23. Although the gold metal (Au) interacted with ARG8, it was unfavorable. Collectively, these interactions stabilize compound **4** within the binding pocket, resulting in a strong binding affinity. Compound **2** was also shown to interact with ASP25, ALA28, and ARG8, forming conventional hydrogen bonds in addition to π–alkyl and alkyl interactions with other binding pocket residues. Similarly, Au was shown to engage in an unfavorable interaction with ARG8. The binding of compound **2**, on the other hand, involved van der Waals, π–alkyl, and alkyl interactions with binding pocket residues. The crucial residues ASP29 and ASP30 were shown to engage in van der Waals interactions with compound **2**, whereas Au did not engage in any visible interaction, which accounts for lower binding affinities than those of other compounds. In general, the three primary metal complexes (compounds **2, 3**, and **4**) displayed interactions with comparable residues to darunavir, along with additional robust interactions with adjacent binding pocket residues. These supplementary interactions have the potential to bolster their binding affinity, as indicated by the docking calculation.

#### Exploring the conformational dynamics of HIV protease upon the binding of metal complexes

**FIGURE 4:**
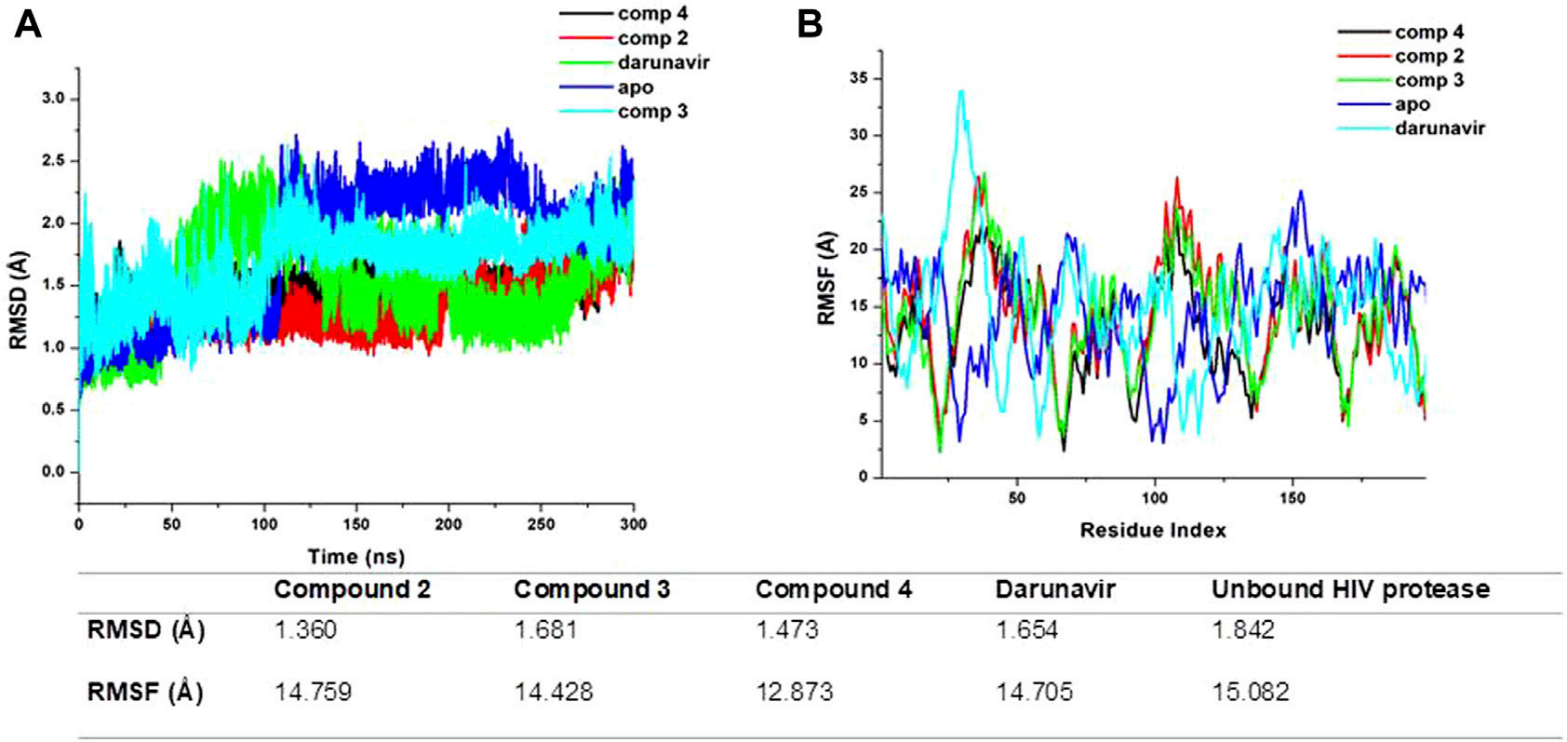
**(A)** Comparative RMSD plots of unbound HIV protease (apo) and its complexed conformation when bound to compounds **2, 3**, and **4** and darunavir. BSA with or without PVP over a 100-ns simulation. **(B)** Comparative RMSF plots of unbound HIV protease (apo) and its complexed conformation when bound to compounds **2, 3**, and **4** and darunavir. BSA with or without PVP over a 100-ns simulation. The table shows average RMSD and RMSF estimations of simulated models over 300 ns.

To elucidate the possible binding mechanism of the compounds toward the HIV protease, we assessed the impact of the binding of the top docked compounds (**2**, **3**, and **4**) by calculating the RMSD and RMSF of C-α atoms of HIV protease atoms over a 300-ns MD simulation. Estimating the RMSD and RMSF of individual atoms unravels the conformational or structural changes that could occur upon ligand binding. The therapeutic binding of chemical compounds to biological targets is typically associated with conformational and structural changes in both the ligand and target receptor, which influence the functions of the biological targets (King et al., 2004). As shown in Figure 4, the binding of all the compounds induced a reduction in the average RMSD and RMSF of the bound HIV protease relative to the unbound structures, suggesting that the binding of the compounds imposed some level of stability and decreased the residue flexibility of HIV protease. HIV protease was also shown to assume a more stable and rigid conformation when bound to the metal complexes than when bound to darunavir. The observed stability and decreased flexibility suggest that the binding of the metal complexes imposes structural rigidity, which could consequently impede the known flexibility of HIV protease, particularly its flap regions, a mechanism consistent with the binding mechanism of some known HIV protease inhibitors, such as darunavir. This observed structural dynamics could also consequently influence the binding interaction with active site residues and overall binding affinity as a relatively stable and rigid conformation could ensure the stability of crucial interactions required for the therapeutic modulation of HIV protease. Nonetheless, further studies are recommended to further validate these findings.

### Synthesized metal complexes exhibit favorable binding affinity toward HIV protease

We estimated the binding affinity of the top three synthesized metal complexes to HIV protease using the MM/GBSA-based binding free energy calculation approach (Ghosh et al., 2006). The MM/GBSA method considers several energy contributions, including vdW and electrostatic interactions, polar solvation (PS) energy, and non-polar solvent-accessible surface area energy. The degree of the binding affinity reflects the strength of interactions between the metal complexes and HIV protease and, making it a crucial component of their binding mechanism. The MM/GBSA calculations results, as given in Table 2, returned an estimated −18.81, −20.31, −26.73, and −31.17 kcal/mol for darunavir and compounds **2**, **3**, and **4,** respectively. This result is consistent with the findings from our RMSD and RMSF calculations, in which the stable and rigid conformation observed in the metal complex-bound structures could have influenced the formation of relatively stable interactions to enhance binding affinities. The MM/GBSA calculations provide additional molecular insights into the therapeutic potential of the synthesized metal complexes as they show enhanced structural modification of HIV protease.

**TABLE 2:** MMPBSA-based binding free energies of the synthesized metal complexes.

Compounds	Energy components (kcal/mol)				
	${\Delta E}_{\text{vdw}}$	${\Delta E}_{\text{ele}}$	${\Delta G}_{\text{gas}}$	${\Delta G}_{\text{sol}}$	${\Delta G}_{\text{bind}}$
**2**	−42.75 ± 0.06	−4.16 ± 0.12	−46.61 ± 0.13	19.61 ± 0.11	−20.31 ± 0.08
**3**	−40.86 ± 4.39	−3.86 ± 0.11	−46.91 ± 0.11	17.98 ± 0.07	−26.73 ± 0.14
**4**	−60.73 ± 0.07	−6.72 ± 0.01	−65.18 ± 0.02	28.46 ± 0.02	−31.17 ± 0.31
**Darunavir**	−37.78 ± 0.30	−2.97 ± 0.21	−38.16 ± 0.05	16.63 ± 0.13	−18.81 ± 0.15

**ΔE**_**ele**_, electrostatic energy; **ΔE**_**vdW**_, van der Waals energy; **ΔG**_**bind**_, total binding free energy; **ΔG**_**sol**_, solvation free energy; **ΔG**, gas phase free energy.

## Conclusion

A series of new gold(I) thiolate derivatives and three bimetallic complexes with amino phosphines and thiocarbohydrates as auxiliary ligands were synthesized and characterized. The structures and purity of complexes **1**–**11** were determined by NMR spectrometry and micro-analysis. It is believed that these thiocarbohydrate gold(I) complexes may show potential as anti-HIV agents since the composition of metals [gold(I), palladium(II), and platinum(II)], phosphines, and thiocarbohydrates is critical in maintaining the activity of the anti-HIV motifs. Molecular modeling further identified three of the metal complexes as potential HIV protease inhibitors, whose binding was characterized by strong binding affinity interactions with binding pocket residues, and the inhibition of the flexibility of the flap regions of the HIV protease was similar to that of the known HIV protease inhibitor, darunavir. This observation is based on computational simulations using molecular dynamics, which suggest a potential mechanism of action for these compounds. However, it is important to note that these findings are theoretical and have not yet been confirmed through experimental validation. Although our computational results are promising, we did not perform experimental assays to directly demonstrate that the synthesized compounds act as protease inhibitors. Future work will focus on conducting biochemical assays, such as enzyme inhibition assays, to empirically validate the inhibitory activity of these compounds against HIV protease.

## Data Availability

The original contributions presented in the study are included in the article/Supplementary Material;further inquiries can be directed to the corresponding author.

## References

[B1] Adachi M. Ohhara T. Kurihara K. Tamada T. Honjo E. Okazaki N. (2009). Structure of HIV-1 protease in complex with potent inhibitor KNI-272 determined by high-resolution X-ray and neutron crystallography. Proc. Natl. Acad. Sci. U. S. A. 106 (12), 4641–4646. 10.1073/pnas.0809400106 19273847 PMC2660780

[B2] Agoni C. Ramharack P. Soliman M. E. (2018a). Co-inhibition as a strategic therapeutic approach to overcome rifampin resistance in tuberculosis therapy: atomistic insights. Future Med. Chem. 10 (14), 1665–1675. 10.4155/fmc-2017-0197 29957065

[B3] Agoni C. Ramharack P. Soliman M. E. (2018b). Synergistic interplay of the co-administration of rifampin and newly developed anti-TB drug: could it be a promising new line of TB therapy? Comb. Chem. High Throughput Screen. 21 (6), 453–460. 10.2174/1386207321666180716093617 30009705

[B4] Agoni C. Salifu E. Y. Enslin G. Kwofie S. K. Soliman M. E. (2022). Dual‐inhibition of human N-myristoyltransferase subtypes halts common cold pathogenesis: atomistic perspectives from the case of IMP-1088. Chem. Biodivers. 19 (2), e202100748. 10.1002/cbdv.202100748 34936193

[B5] Arrigoni R. Santacroce L. Ballini A. Palese L. L. (2023). AI-aided search for new HIV-1 protease ligands. Biomolecules 13 (5), 858. 10.3390/biom13050858 37238727 PMC10216636

[B6] Beaglehole R. Bonita R. Horton R. Adams C. Alleyne G. Asaria P. (2011). Priority actions for the non-communicable disease crisis. lancet 377 (9775), 1438–1447. 10.1016/s0140-6736(11)60393-0 21474174

[B7] Berendsen H. J. Postma J. P. M. van Gunsteren W. F. DiNola A. Haak J. R. (1984). Molecular dynamics with coupling to an external bath. J. Chem. Phys. 81 (8), 3684–3690. 10.1063/1.448118

[B8] BIOVIA Discovery Studio (2016). Discovery studio modeling environment, release, 4. San Diego: Dassault Systemes BIOVIA.

[B9] Case D. A. Cheatham T. E. Darden T. Gohlke H. Luo R. Merz Jr K. M. (2005). The Amber biomolecular simulation programs. J. Comput. Chem. 26 (16), 1668–1688. 10.1002/jcc.20290 16200636 PMC1989667

[B10] ChemAxon M. S. (2013). Budapest. Hungary Version 6.

[B11] Chuntakaruk H. Hengphasatporn K. Shigeta Y. Aonbangkhen C. Lee V. S. Khotavivattana T. (2024). FMO-guided design of darunavir analogs as HIV-1 protease inhibitors. Sci. Rep. 14 (1), 3639. 10.1038/s41598-024-53940-1 38351065 PMC10864397

[B12] Coetzee J. Gabrielli W. F. Coetzee K. Schuster O. Nogai S. D. Cronje S. (2007). Structural studies of gold (I, II, and III) compounds with pentafluorophenyl and tetrahydrothiophene ligands. Angew. Chem. 119 (14), 2549–2552. 10.1002/ange.200604592 17328088

[B13] Dar A. M. Mir S. (2017). Molecular docking: approaches, types, applications and basic challenges. J. Anal. Bioanal. Tech. 8, 1–7. 10.4172/2155-9872.1000356

[B14] Dassault Systèmes (2016). Biovia, discovery studio modeling environment. San Diego, CA, USA: Dassault Systèmes Biovia.

[B15] Fillat M. F. Gimeno M. C. Laguna A. Latorre E. Ortego L. Villacampa M. D. (2011). Synthesis, structure and bactericide activity of (Aminophosphane)gold(I) thiolate complexes. Eur. J. Inorg. Chem. 2011, 1487–1495. 10.1002/ejic.201001195

[B16] Fonteh P. Meyer D. (2009). Novel gold (I) phosphine compounds inhibit HIV-1 enzymes. Metallomics 1 (5), 427–433. 10.1039/b909036c 21305147

[B17] Genheden S. Ryde U. (2015). The MM/PBSA and MM/GBSA methods to estimate ligand-binding affinities. Expert Opin. Drug Discov. 10 (5), 449–461. 10.1517/17460441.2015.1032936 25835573 PMC4487606

[B18] Ghosh A. K. (2023). Four decades of continuing innovations in the development of antiretroviral therapy for HIV/AIDS: progress to date and future challenges. Glob. Health and Med. 5 (4), 194–198. 10.35772/ghm.2023.01013 37655189 PMC10461327

[B19] Ghosh A. K. Osswald H. L. Prato G. (2016). Recent progress in the development of HIV-1 protease inhibitors for the treatment of HIV/AIDS. J. Med. Chem. 59 (11), 5172–5208. 10.1021/acs.jmedchem.5b01697 26799988 PMC5598487

[B20] Ghosh A. K. Ramu Sridhar P. Kumaragurubaran N. Koh Y. Weber I. T. Mitsuya H. (2006). Bis-tetrahydrofuran: a privileged ligand for darunavir and a new generation of HIV protease inhibitors that combat drug resistance. ChemMedChem 1 (9). 939. 10.1002/chin.200648245 16927344

[B21] Hanwell M. D. Curtis D. E. Lonie D. C. Vandermeersch T. Zurek E. Hutchison G. R. (2012). Avogadro: an advanced semantic chemical editor, visualization, and analysis platform. J. Cheminformatics 4, 1–17. 10.1186/1758-2946-4-17 PMC3542060 22889332

[B22] Hofer F. Kraml J. Kahler U. Kamenik A. S. Liedl K. R. (2020). Catalytic site pKa values of aspartic, cysteine, and serine proteases: constant pH MD simulations. J. Chem. Inf. Model 60 (6), 3030–3042. 10.1021/acs.jcim.0c00190 32348143 PMC7312390

[B23] Hussein M. Molina M. A. Berkhout B. Herrera-Carrillo E. (2023). A CRISPR-cas cure for HIV/AIDS. Int. J. Mol. Sci. 24 (2), 1563. 10.3390/ijms24021563 36675077 PMC9863116

[B24] Jiang X. Ahmed M. Deng Z. Narain R. (2009). Biotinylated glyco-functionalized quantum dots: synthesis, characterization, and cytotoxicity studies. Bioconjugate Chem. 20 (5), 994–1001. 10.1021/bc800566f 19402705

[B25] King N. M. Prabu-Jeyabalan M. Nalivaika E. A. Wigerinck P. de M. P. Béthune C. A. (2004). Structural and thermodynamic basis for the binding of TMC114, a next-generation human immunodeficiency Virus type 1 protease inhibitor. J. Virology 78, 12012–12021. 10.1128/jvi.78.21.12012-12021.2004 15479840 PMC523255

[B26] Kongsted J. Söderhjelm P. Ryde U. (2009). How accurate are continuum solvation models for drug-like molecules? J. Comput. Aided Mol. Des. 23, 395–409. 10.1007/s10822-009-9271-6 19444622

[B27] Kožíšek M. Lepšík M. Grantz Šašková K. Brynda J. Konvalinka J. Rezáčová P. (2014). Thermodynamic and structural analysis of HIV protease resistance to darunavir–analysis of heavily mutated patient‐derived HIV‐1 proteases. FEBS J. 281 (7), 1834–1847. 10.1111/febs.12743 24785545

[B28] Kräutler V. Van Gunsteren W. F. Hünenberger P. H. (2001). A fast SHAKE algorithm to solve distance constraint equations for small molecules in molecular dynamics simulations. J. Comput. Chem. 22 (5), 501–508. 10.1002/1096-987x(20010415)22:5<501::aid-jcc1021>3.0.co;2-v

[B29] Le Grand S. Götz A. W. Walker R. C. (2013). SPFP: speed without compromise—a mixed precision model for GPU accelerated molecular dynamics simulations. Comput. Phys. Commun. 184 (2), 374–380. 10.1016/j.cpc.2012.09.022

[B30] Levitt N. S. Steyn K. Dave J. Bradshaw D. (2011). Chronic noncommunicable diseases and HIV-AIDS on a collision course: relevance for health care delivery, particularly in low-resource settings—insights from South Africa. Am. J. Clin. Nutr. 94 (6), 1690S–1696S. 10.3945/ajcn.111.019075 22089433 PMC3226022

[B31] Lin X. Li X. Lin X. (2020). A review on applications of computational methods in drug screening and design. Molecules 25 (6), 1375. 10.3390/molecules25061375 32197324 PMC7144386

[B32] Maier J. A. Martinez C. Kasavajhala K. Wickstrom L. Hauser K. E. Simmerling C. (2015). ff14SB: improving the accuracy of protein side chain and backbone parameters from ff99SB. J. Chem. Theory Comput. 11 (8), 3696–3713. 10.1021/acs.jctc.5b00255 26574453 PMC4821407

[B33] Miller B. R. McGee T. D. Jr. Swails J. M. Homeyer N. Gohlke H. Roitberg A. E. (2012). MMPBSA. py: an efficient program for end-state free energy calculations. J. Chem. theory Comput. 8 (9), 3314–3321. 10.1021/ct300418h 26605738

[B34] Mohammadnejad S. Provis J. L. van Deventer J. S. J. (2015). Computational modelling of gold complexes using density functional theory. Comput. Theor. Chem. 1073, 45–54. 10.1016/j.comptc.2015.09.005

[B35] Olotu F. A. Agoni C. Adeniji E. Abdullahi M. Soliman M. E. (2019). Probing gallate-mediated selectivity and high-affinity binding of epigallocatechin gallate: a way-forward in the design of selective inhibitors for anti-apoptotic bcl-2 proteins. Appl. Biochem. Biotechnol. 187, 1061–1080. 10.1007/s12010-018-2863-7 30155742

[B36] Pettersen E. F. Goddard T. D. Huang C. C. Couch G. S. Greenblatt D. M. Meng E. C. (2004). UCSF Chimera—a visualization system for exploratory research and analysis. J. Comput. Chem. 25 (13), 1605–1612. 10.1002/jcc.20084 15264254

[B37] Prieto-Martínez F. D. Arciniega M. Medina-Franco J. L. (2018). Molecular docking: current advances and challenges. TIP Revista Especializada en Ciencias Químico-Biológicas 25. 10.22201/fesz.23958723e.2018.0.143

[B38] Privat C. Madurga S. Mas F. Rubio-Martínez J. (2020). On the use of the discrete constant pH molecular dynamics to describe the conformational space of peptides. Polym. (Basel) 13 (1), 99. 10.3390/polym13010099 PMC7795291 33383731

[B39] Ramharack P. Salifu E. Y. Agoni C. (2023). Dual-target Mycobacterium tuberculosis inhibition: insights into the molecular mechanism of antifolate drugs. Int. J. Mol. Sci., 24(18), 14021, 10.3390/ijms241814021 37762327 PMC10530724

[B40] Rastelli G. Del Rio A. Degliesposti G. Sgobba M. (2010). Fast and accurate predictions of binding free energies using MM-PBSA and MM-GBSA. J. Comput. Chem. 31 (4), 797–810. 10.1002/jcc.21372 19569205

[B41] Roe D. R. Cheatham III T. E. (2013). PTRAJ a*nd* CPPTRAJ: software for processing and analysis of molecular dynamics trajectory data. J. Chem. theory Comput. 9 (7), 3084–3095. 10.1021/ct400341p 26583988

[B42] Salomon-Ferrer R. Götz A. W. Poole D. Le S. Grand R. C. (2013). Routine microsecond molecular dynamics simulations with AMBER on GPUs. 2. Explicit solvent particle mesh Ewald. J. Chem. Theory Comput. 9 (9), 3878–3888. 10.1021/ct400314y 26592383

[B43] Schauperl M. Nerenberg P. S. Jang H. Wang L. P. Bayly C. I. Mobley D. L. (2020). Non-bonded force field model with advanced restrained electrostatic potential charges (RESP2). Commun. Chem. 3 (44), 44–11. 10.1038/s42004-020-0291-4 34136662 PMC8204736

[B44] Seifert E. (2014). OriginPro 9.1: scientific data analysis and graphing software-software review. J. Chem. Information Model. 54 (5), 1552. 10.1021/ci500161d 24702057

[B45] Trott O. Olson A. J. (2010). AutoDock Vina: improving the speed and accuracy of docking with a new scoring function, efficient optimization, and multithreading. J. Comput. Chem. 31 (2), 455–461. 10.1002/jcc.21334 19499576 PMC3041641

[B46] Unaids D. Sheet A. F. (2021). Available at: https://www.unaids.org/sites/default/files/media_asset.UNAIDS_FactSheet_en.pdf (Accessed April 7, 2022).

[B47] Wang E. Liu H. Wang J. Weng G. Sun H. Wang Z. (2020). Development and evaluation of MM/GBSA based on a variable dielectric GB model for predicting protein–ligand binding affinities. J. Chem. Inf. Model. 60 (11), 5353–5365. 10.1021/acs.jcim.0c00024 32175734

